# Risk Perception of COVID-19, Meaning-Based Resources and Psychological Well-Being amongst Healthcare Personnel: The Mediating Role of Coping

**DOI:** 10.3390/jcm9103225

**Published:** 2020-10-08

**Authors:** Dariusz Krok, Beata Zarzycka

**Affiliations:** 1 Institute of Psychology, University of Opole, 45-040 Opole, Poland; dkrok@uni.opole.pl; 2 Institute of Psychology, John Paul II Catholic University of Lublin, 20-950 Lublin, Poland

**Keywords:** risk perception of COVID-19, meaning-based resources, psychological well-being, healthcare personnel

## Abstract

The well-being of healthcare personnel during the COVID-19 pandemic depends on the ways in which they perceive the threat posed by the virus, personal resources, and coping abilities. The current study aims to examine the mediating role of coping strategies in the relationship between risk perception of COVID-19 and psychological well-being, as well as the relationship between meaning-based resources and psychological well-being amongst healthcare personnel in southern Poland. Two hundred and twenty-six healthcare personnel who worked in hospitals, outpatient clinics, and medical laboratories during the first few months of the coronavirus pandemic (March–May 2020) filled in questionnaires measuring risk perception of COVID-19, meaning-based resources, coping, and psychological well-being. The results demonstrate that risk perception was negatively related to psychological well-being, whereas meaning-based resources were positively associated with well-being. Two coping strategies—problem-focused and meaning-focused coping—mediated the relationship between risk perception and psychological well-being as well as the relationship between meaning-based resources and psychological well-being. This indicates that perception processes and personal factors do not directly influence healthcare personnel’s psychological well-being, but rather they do indirectly through coping processes.

## 1. Introduction

The highly infectious coronavirus SARS-CoV-2 caused an epidemic of acute respiratory syndrome (COVID-19). Between January and April 2020, the epidemic turned into a global pandemic, having spread to most countries around the world [1]. In March 2020, the World Health Organization made the assessment that COVID-19 was as a pandemic. In Poland, the COVID-19 pandemic began on 4 March 2020, and on 5 October 2020 the number of people infected with SARS-CoV-2 had risen to 102,080; of those, 2659 died. The outbreak of the coronavirus disease caused intense stress amongst the public in Poland, and healthcare personnel were one of the most affected groups. Since the outbreak, healthcare personnel have found themselves on the frontline in combating COVID-19, working with an increased workload in terms of working hours and patient numbers and facing the highest risk of infection. Thus, operating under such conditions may have contributed to increased psychological stress, with immediate and perhaps long-term psychological consequences [2].

There have as yet been few studies on the psychological effects of working in a healthcare setting around COVID-19. Research conducted in China showed that after the COVID-19 outbreak, medical staff experienced emotional stress, depression, insomnia, and anxiety [2,3,4]. Nurses and frontline healthcare workers reported more severe degrees of all measurements of mental health symptoms than other healthcare personnel [3]. Although the psychological effects of outbreaks of COVID-19 disease on healthcare personnel have been demonstrated [2,3,4], little is known about the combined roles of risk perception and individual resources for well-being, and the mediating effects of coping on their relationships. These factors have also not been studied together in healthcare workers. Thus, it is important that these relationships are examined, because the findings may shed more light on specific factors that contribute significantly to the creation of new intervention programs (e.g., meaning-based approaches that address existential distress and psychological well-being). Healthcare personnel need to protect the well-being of themselves and their colleagues to avoid adverse outcomes for both healthcare workers and patients.

### 1.1. Risk Perception, Meaning-Based Resources, and Psychological Well-Being

Appraisal-based and resource-based stress theories have been successfully employed in predicting a range of stress outcomes in health contexts [5,6]. Appraisal-based theories, which include the protection motivation theory (PMT), describe how people assess a threat’s probability [7], whereas resource-based theories, amongst which the conservation of resources (COR) theory is one of the most prominent, indicate that personal resources enable people to cope effectively with the threat [8].

Protection motivation theory has been successfully applied in the context of health threats to explain the effects of fear on people’s reactions and attitudes [7]. It emphasizes the importance of risk perception, which consists of three components: (1) perceived risk of contracting, which is the person’s expectation of being exposed to the threat, such as being infected by COVID-19; (2) fear, which plays an indirect role in threat appraisal by affecting the estimation of the danger’s severity; and (3) perceived threat, which is the person’s estimation of how harmful the consequences of the threat would be to objects they value if the threat were to actually occur (e.g., the judgment that a COVID-19 infection would harm valued things, such as personal health or the health of other people) [9]. Previous studies indicated that perceived risk was negatively related to well-being during the outbreak of severe acute respiratory syndrome (SARS). Research conducted on SARS survivors demonstrated that their subjective interpretation of the infection was related to the level of psychological adjustment measured in terms of emotional distress and perceived health [10]. The few studies carried out so far during the outbreak of COVID-19 amongst the Chinese public revealed that perceived threat was related to a number of undesirable emotional reactions (e.g., an increase in negative emotion) [3] and that self-control moderates the association between perceived threat and mental health problems amongst the Chinese public [11]. Both studies led to the conclusion that the perceived threat of COVID-19 may negatively influence mental health outcomes.

Conservation of resources theory begins with the tenet that individuals strive to obtain, retain, foster, and protect personal resources, defined as “those entities that either are centrally valued in their own right, or act as means to obtain centrally valued ends” [8] (p. 307). The fit of personal resources with external demands determines the direction of stress response and resultant outcomes [5]. Research has demonstrated that resources influence people’s abilities to impact their environments successfully, and thus they are typically linked to well-being, positive coping, and global resistance to stress [12]. The studies examining relationships between resources and well-being involve a number of distinctive types of resource [8], among which meaning based resources, e.g., meaning in life (MIL) and existential mattering, are widely studied.

Meaning in life (MIL) has been regarded as a key resource for both coping processes and psychological well-being [13,14]. Meaning in life has been defined as “the extent to which one’s life is experienced as making sense, as being directed and motivated by valued goals, and as mattering in the world” [15] (p. 205). Considering the predictive role of MIL on people’s psychological health [16,17,18], examining MIL levels amongst healthcare staff is relevant for many reasons. Meaning in life in healthcare settings plays a vital role in the construction of the individual’s identity, and it can provide one of the main sources of inner harmony [19]. In addition, MIL can noticeably influence the ways in which healthcare workers deal with stress and maintain their professional efficiency [20]. Given that the pandemic is considered a prolonged stressful condition, particularly for those who are in the front line in the fight against the virus, the availability of meaning-based resources would help to manage such stress successfully.

Another factor related to meaning-based resources is existential mattering, which has received a considerable deal of empirical attention in recent years [21,22]. This may be described as one’s experiences of value, worth, and transcending everyday life conditions, and is regarded as a core dimension of meaning structures in addition to more commonly accepted significance and purpose [23]. Research has demonstrated that mattering is beneficial to developing self-identity, self-concept, sense of belonging, and understanding one’s purpose in life [21]. By developing a sense of mattering in their lives, individuals can gain an awareness of being able to make a difference in the world and to lead a valuable life. Mattering was also found to play a crucial role in psychological well-being and health amongst college and university students [24].

The inclusion of meaning-based resources noticeably influenced psychological models of stress and coping due to the internal structures of purpose and value embedded in the resources. They tend to affect the ways in which important events are perceived as well as how these events are managed [20,25]. Being related to different measures of well-being, they are also likely to affect one’s coping responses to stressful events (e.g., public health threats or unpredictable infections).

### 1.2. Coping Strategies as Mediators

Coping is defined as an effort to manage demands that are appraised as exceeding the resources of the person [26]. Two classes of coping are commonly identified: problem-focused and emotion-focused. The former involves efforts to obtain information about what to do as well as how to alter the stressful situation (e.g., information seeking, planning, or taking action). The latter involves efforts to regulate the emotional distress associated with the situation (e.g., seeking emotional support from others or by behavioral disengagement) [27]. More recently, meaning-focused coping—involving changing the appraised meaning of a situation to make it more consistent with individuals’ beliefs and goals—was posited [28,29]. Research demonstrated that the three coping strategies often work in tandem; the regulation of anxiety and fear (emotion-focused coping) enables the person to focus on taking a decision (problem-focused coping), or the cognitive restructuring (problem-focused coping) can be guided by underlying values and goals (meaning-focused coping) [29,30]. In addition, different coping strategies appear to be beneficial depending on the particular situation and context, e.g., problem-focused coping was found to be predominantly helpful in high controllability situations, while emotion-focused coping was more effective under low controllability conditions [31].

Previous studies confirmed that coping played a mediating role in relationships between various forms of risk perception and well-being measures. Coping was found to mediate the relationship of ecological risk with depressive symptoms for African Americans [32]. Avoidant coping was a mediator in associations between a form of risk rejection (i.e., evaluative concerns about perfectionism) and distress [33]. In addition, risk perception tended to influence coping strategies in people who had experienced either natural or industrial catastrophe [34]. Environmental risk perception was also confirmed as a predictor of coping behaviors [35].

Coping played an important role in relationships between personal resources and well-being. Coping self-efficacy mediated the associations between self-esteem and optimism and distress experienced in the aftermath of the 1999 earthquake in Turkey [36]. Coping focused on change mediated the relationship between personal resources conceptualized as psychological capital and well-being [37]. Meaning in life was also reported to influence coping behavior after disaster [20] and to be a predictor of the appropriate use of coping and stress management resources at work [19].

These results taken together imply that individuals may use coping in relation to their appraisals of stressful events and personal resources, which will subsequently influence well-being measures [37,38]. There can be also differential predictions for the different strategies as risk perception and personal resources are differently related to the coping strategies. However thus far, no studies have examined the mediating role of coping between risk perception and psychological well-being as well as between meaning-based resources and psychological well-being in the context of the COVID-19 pandemic. A German sample showed that the risk perception of being infected by COVID-19 was higher in women than men and higher in older people than younger people, in conjunction with a more frequent use of problem-focused strategies. The relationship between COVID-19 risk perception and coping strategies was not analyzed [39].

### 1.3. The Present Study

The present study aims to elucidate the mediating contribution of coping strategies in the association between risk perception of COVID-19 and psychological well-being as well as the association between meaning-based resources and psychological well-being amongst healthcare personnel. Based on the research presented above, three hypotheses were formulated. First, we hypothesized that risk perception of COVID-19 will be negatively and directly associated with psychological well-being, while meaning-based resources will be positively and directly associated with the psychological well-being of healthcare personnel on the frontline in combating COVID-19 (Hypothesis 1). Second, given that the relationship between the individual’s appraisal of stressful events and psychological outcomes depends on the way they cope with challenging situations [20,40], we hypothesized that coping strategies will mediate relationships between risk perception of COVID-19 and psychological well-being (Hypothesis 2). Third, given that personal resources play an important role in shaping coping behavior, which consequently determines psychological adjustment [37,38], we hypothesized that coping strategies would mediate the meaning-based resources and psychological well-being link (Hypothesis 3). Table 1 shows the summary of key constructs included in the study.

## 2. Experimental Section

### 2.1. Participants and Procedure

In order to generate a representative sample of healthcare workers, quota sampling was used. Quota sampling is a non-probabilistic sampling method in which the population of healthcare workers was divided in equally exclusive subgroups. First, we selected the subgroups of healthcare workers with regard to five criteria: gender, age, types of healthcare professions, years of service, and a level of education. Second, we identified the proportions of these subgroups and then selected subjects from the various subgroups on a basis of these proportions. The aim was to assemble a sample that would have the same proportions of individuals and be representative of the entire population of healthcare workers with respect to the abovementioned characteristics. The final sample consisted of 226 healthcare personnel working in hospitals, outpatient clinics, and medical laboratories in southern Poland during the first few months of the coronavirus pandemic (March–May 2020), and who were consequently exposed to the associated health risks. The group comprised the following professions: doctors (*n* = 51), nurses (*n* = 113), laboratory technicians (*n* = 22), aides and assistants (*n* = 29), and physiotherapists (*n* = 11). Of the participants, 58.8% were female and 41.2% were male. Their average age was 37.36 (SD = 13.59). All were employed either full-time or part-time.

Participants were recruited in hospitals, outpatient clinics, and medical laboratories, with the aim of acquiring a representative sample of healthcare personnel. They were provided with information regarding the purpose and rules of participation and were asked to fill in an online or printed version of the questionnaire. A research assistant was available in case any additional information was requested. The University Ethics Board accepted the research material and procedure.

### 2.2. Measures

The following scales and questionnaires were used in the study. The summary scores for the study variables were calculated by summing responses to each item.

#### 2.2.1. Risk of Contracting COVID-19

Drawing on the conceptualization of risk perception proposed by Grothmann and Reusswig, who defined it as the extent to which an individual “assesses a threat’s probability and damage potential” [9] (p. 104), the risk of contracting COVID-19 scale was developed [41]. The scale comprises 6 items rated from 1 (strongly disagree) to 5 (strongly agree). The sample items are: “Getting infected with coronavirus threatens my health” and “I am worried that I may become infected with coronavirus”. A high score reflects a stronger perceived probability of contracting coronavirus. The Cronbach’s α reliability for the sample was 0.85.

#### 2.2.2. Fear of COVID-19

The level of fear experienced by individuals in the context of coronavirus was measured with 6 items on a scale ranging from 1 (strongly disagree) to 5 (strongly agree). They represent emotional reactions of anxiety and apprehension caused by the coronavirus pandemic [42]. The sample items are: “I am afraid of serious health complications caused by coronavirus” and “I fear an extended hospital stay in case of being infected by coronavirus”. The Cronbach’s α reliability for the sample was 0.84.

#### 2.2.3. Perceived Threat of COVID-19

Perceptions of threat severity of coronavirus were evaluated with the perceived threat of COVID-19 scale [41], which includes 6 items rated from 1 (strongly disagree) to 5 (strongly agree). The scale measures perceived threat severity of coronavirus that pertains to the negative personal, societal, and economic consequences people associate with the coronavirus pandemic. The sample items include: “Coronavirus is a serious threat to people” and “The coronavirus pandemic has a damaging impact on the economic situation of our country”. The Cronbach’s α reliability for the sample was 0.81.

#### 2.2.4. Meaning in Life

Meaning in life was measured with the meaning in life questionnaire [43], which consists of 10 items rated from 1 (strongly disagree) to 7 (strongly agree). The questionnaire includes two dimensions: presence of meaning in life and search for meaning in life. As the aim of our study focuses primarily on the present characteristics of meaning in life experienced by participants, we only used the presence subscale that assesses how much people perceive their lives as meaningful. The Cronbach’s α reliability for the sample was 0.83.

#### 2.2.5. Existential Mattering

Existential mattering, which is regarded as the strongest indicator of meaning judgments, was measured with 6 items on a scale ranging from 1 (strongly disagree) to 5 (strongly agree). They assessed existential meaning, which is understood to be the conviction that one’s life is significant, important, and valuable in the world [15,23]. The sample items include: “I am sure my life matters” and “I see my life as existentially significant”. The Cronbach’s α reliability for the sample was 0.80.

#### 2.2.6. Coping

Coping was measured with the coping questionnaire [44], a 37-item instrument that assesses three coping strategies: problem-focused coping, emotion-focused coping, and meaning-focused coping. Participants respond to items on a Likert scale ranging from 1 (not at all) to 5 (very much). The total score was calculated by summing responses to each item. The Cronbach’s α reliability for the sample was 0.84 for problem-focused coping, 0.83 for emotion-focused coping, and 0.87 for meaning-focused coping.

#### 2.2.7. Psychological Well-Being

Psychological well-being was measured with the psychological well-being Scale [45] adapted for Polish by Karas and Cieciuch [46]. The short version contains 18 items rated from 1 (strongly disagree) to 6 (strongly agree). The scale comprises six dimensions: self-acceptance, positive relations with others, purpose in life, environmental mastery, personal growth, and autonomy, the score of which gives the total result. Due to the statistical analysis employed in the present study, we only used the total score, which was calculated by summing responses to each item. The Cronbach’s α reliability for the sample was 0.84.

### 2.3. Data Analysis

Pearson’s correlations were calculated to examine the relationships among the variables. Then, structural equation modelling (SEM) was used to estimate the statistical models (Amos 21 SPSS, an IBM Company: Chicago, IL, USA) [47]. In accordance with the rules of SEM, we first validated the measurement model by confirmatory factor analyses (CFA) and then we tested the structural relationship between measured variables and latent constructs. The goodness of fit of the model was evaluated by applying different indices: χ^2^ statistic, the standardized root-mean-square residual (SRMR), root-mean-square error of approximation (RMSEA), and three modification indices—goodness of fit index (GFI), Tucker Lewis index (TLI), and comparative fit index (CFI). We were removing non-significant parameters and non-significant paths of the original model one at a time in relation to the modification indices to improve the model fit (Table S1, Supplementary Material) [48]. In addition, the modifications were to improve the model fit as long as they were corroborated by the theory. Bootstrapping was used to estimate direct and indirect effects (samples = 5000; 95% bias-corrected confidence intervals; standardized coefficients were presented). This approach enabled us to examine the relationship between risk perception of COVID-19 and psychological well-being as well as the relationship between meaning-based resources and psychological well-being.

## 3. Results

### 3.1. Descriptive Statistics and Correlations Amongst Variables

First, the correlations among risk perception of the COVID-19, meaning-based resources, and psychological well-being were tested. They are presented in Table 2.

Risk of contracting COVID-19 and fear of COVID-19 were positively related to problem-focused coping, emotion-focused coping and meaning-focused coping, and negatively related to psychological well-being. Perceived threat of COVID-19 positively correlated with emotion-focused coping and meaning-focused coping but was negatively associated with psychological well-being. Interestingly, there was no significant correlation between the factors forming risk perception of COVID-19 (risk of contracting, fear, and perceived threat) and meaning-based resources (meaning in life and existential mattering). In contrast, meaning in life and existential mattering were positively related to all coping strategies and psychological well-being. Problem-focused coping, emotion-focused coping, and meaning-focused coping were also positively connected to psychological well-being.

### 3.2. Direct and Indirect Effects of Risk Perception of COVID-19 and Meaning-Based Resources on Psychological Well-Being: Mediating Effects of Coping

The theoretical model constructed on the basis of previous findings assumed that the relationship of risk perception of COVID-19 and meaning-based resources with psychological well-being could be mediated by coping strategies. To verify these assumptions, structured equation modelling (SEM) analysis and a bootstrapping procedure were applied, as recommended by Preacher and Hayes [47].

#### 3.2.1. Measurement Model

First, the measurement model, including loadings of indicators, was tested in relation to their corresponding latent variables by confirmatory factor analyses (CFA). Two latent factors were specified: (1) risk perception of COVID-19 that represents the individual’s negatively-oriented attitude, encompassing risk of contracting, fear, and perceived threat, and (2) meaning-based resources that comprise meaning in life and existential mattering. The CFA results, including two latent factors and five observed variables, confirmed a very satisfactory fit to the data: χ^2^ (*n* = 226) = 7.16, *p* < 0.001; GFI = 0.98; CFI = 0.98; NFI = 0.95; RMSEA = 0.04; SRMR = 0.02. The factor loadings obtained for the latent variables’ indicators reached statistical significance (*p* < 0.001) (Table 3).

#### 3.2.2. Structural Model

**Figure 1:**
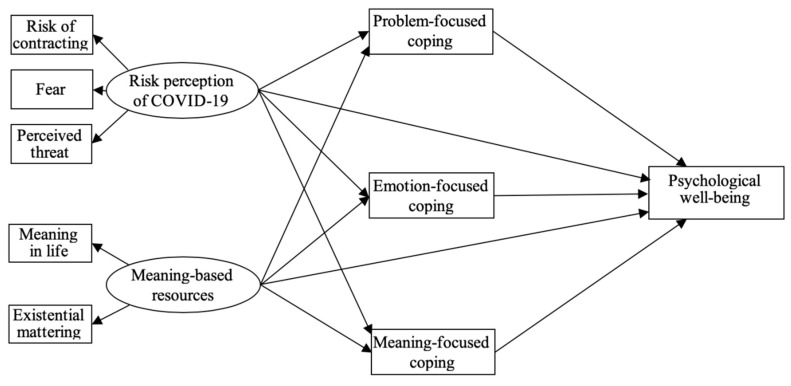
The theoretical model of the relations among risk perception of COVID-19, meaning-based resources, coping, and psychological well-being.

Drawing on earlier correlational results, the hypothesized mediation model including direct and indirect paths between two independent variables (risk perception of COVID-19 and meaning-based resources), three mediating variables (coping strategies), and the dependent variable (psychological well-being) was tested (Figure 1). Gender and age were also controlled in our structural analysis. In addition, each mediator was estimated independently, which generated more accurate mediating effects.

Structured equation modelling analysis revealed that model 1, which included three mediators and direct paths from risk perception of COVID-19 and meaning-based resources to psychological well-being had rather an unsatisfactory fit with the data: χ^2^ (20, *n* = 226) = 59.84, *p* < 0.001; GFI = 0.88; CFI = 0.83; NFI = 0.78; RMSEA = 0.09. Furthermore, some path coefficients were nonsignificant.

**Figure 2:**
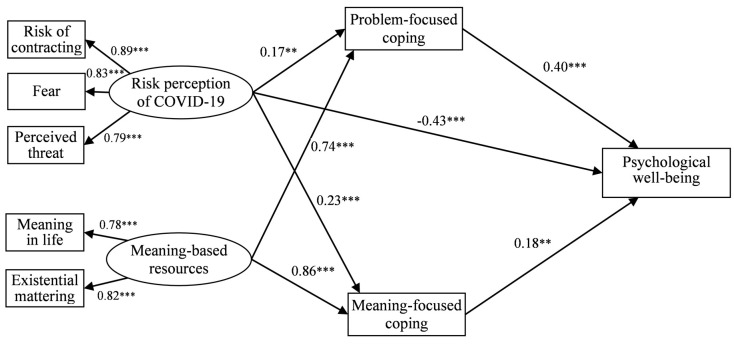
The final mediating model of the relations among risk perception of COVID-19, meaning-based resources, coping, and psychological well-being (standardized coefficients). ** *p* < 0.01; *** *p* < 0.001.

The model was thus modified to improve fit in accordance with the modification procedures (the order in which variables and paths were removed is included in a supplement). Emotion-focused coping and nonsignificant paths from risk perception of COVID-19 and meaning-based resources to psychological well-being were deleted. As a consequence, the final model turned out to have significant improvements and a satisfactory fit: χ^2^ (15, *n* = 226) = 38.36, *p* < 0.001; GFI = 0.95; CFI = 0.93; NFI = 0.92; RMSEA = 0.04 (model 2, Figure 2).

The final model contained one statistically significant direct effect (β = −0.43) from risk perception of COVID-19 to psychological well-being, which indicates that higher risk perception was related to a lower level of psychological well-being. In addition, problem-focused and meaning-focused coping accounted for indirect effects. Risk perception of COVID-19 had indirect relationships with psychological well-being through the two aforementioned coping strategies. The positive directions of those paths suggested that higher risk perception was related to more frequent use of problem- and meaning-focused coping, which again was related to a higher level of psychological well-being. Analogously, there was an indirect relationship between meaning-based resources and psychological well-being through significant positive paths comprising problem-focused coping and meaning-focused coping. More meaning-based resources were associated with more frequent use of problem- and meaning-focused coping, which was then associated with greater psychological well-being.

The bootstrapping procedure was applied to examine the mediating effects of problem- and meaning-focused coping on the association between risk perception of COVID-19 and psychological well-being as well as the association between meaning-based resources and psychological well-being (samples = 5000; 95% bias-corrected confidence intervals [47]) (Table 4).

The results of mediation analysis revealed that problem- and meaning-focused coping mediated the relationship between risk perception of COVID-19 and psychological well-being as well as the relationship between meaning-based resources and psychological well-being. Interestingly, despite conceptual differences between risk perception and meaning-based resources, the mediating effects showed a similar pattern for both paths. Risk perception and meaning-based resources exerted significant indirect effects on the sphere of well-being via problem-focused and meaning-focused coping strategies. The total effects, which denote the sum of the direct and indirect effects, were significant for both paths, which implies therefore that problem-focused coping and meaning-focused coping function in the form of simultaneous mediators.

In addition, we decided to test whether those two indirect effects are significantly different (i.e., whether one or the other of the mediating effects is stronger). The result demonstrates that the mediation for the meaning-based resources–coping–psychological well-being path was significantly stronger than for the risk perception–coping–psychological well-being path (effect = 0.33; SE = 0.06; 95% CI = 0.05 to 0.41).

## 4. Discussion

The purpose of this study was to examine risk perception of COVID-19 infection and meaning-based resources in relation to coping strategies and psychological well-being amongst healthcare personnel in southern Poland during the COVID-19 outbreak between March and June 2020. We found that risk perception of COVID-19 infection—risk of contracting, fear, and perceived threat of COVID-19—negatively correlated with psychological well-being, whereas meaning-based resources (i.e., MIL and existential mattering) correlated positively with well-being. Moreover, both risk perception of COVID-19 infection and meaning-based resources were related to psychological well-being through the mediating effect of coping strategies.

As was hypothesized, risk perception, which reflects how healthcare personnel assess the probability of COVID-19 infection negatively correlated with psychological well-being. These findings are in line with previous studies that demonstrated perceived risk as a predictor of emotional distress amongst SARS survivors during the SARs epidemic in Hong Kong [10], as well as negative emotions amongst the Chinese population during the COVID-19 pandemic [49]. Our findings complement previous research in two important ways. First, both Cheng et al. [10] and Li et al. [49] studies were carried out amongst the Chinese general population, yet the health workers in Europe are exposed to a greater risk of infection, because of the rapid spread of COVID-19 and the sudden huge influx of patients. Second, SEM analysis revealed that the direct relationship between risk perception and healthcare personnel’s well-being remained significant, even after controlling for the mediating effect of coping strategies. This implies the relatively strong character of the relationship between the way in which healthcare personnel interpret risk of contracting, fear, and perceived threat of COVID-19 infection and their psychological well-being.

In contrast, meaning-based resources positively correlated with psychological well-being. Thus, having more meaning-based resources was associated with higher well-being amongst healthcare personnel, which, in turn, can be used to deal with the negative psychological consequences of COVID-19. Healthcare workers who have a strong sense of purpose and value can more efficiently interpret and reorganize daily experiences, identify significant aspects of their life, and constructively pursue their aims. This finding corresponds with previous studies that indicated the predictive role of MIL on people’s well-being [16,17,18] as well as the predictive role of MIL on the construction of individual identity amongst medical staff [19]. However, the direct relationship between meaning-based resources with psychological well-being was not obtained in the SEM analysis, after controlling for coping strategies. Given that SEM is considered to deliver more accurate results than correlations [50], these findings support an indirect rather than a direct relationship between meaning-based resources and well-being amongst healthcare personnel. Therefore, the availability of meaning-based resources can strengthen psychological well-being because they successfully trigger an effective use of coping strategies that can be applied to manage stress during the COVID-19 pandemic. The first hypothesis was only partially confirmed.

The main finding in this study was the mediating role played by coping strategies in the relationships between risk perception of the COVID-19 infection and meaning-based resources with healthcare personnel’s psychological well-being. Two coping strategies—problem-focused and meaning-focused—were significant mediators in these relationships. The second and third hypotheses that assumed such relationships were thus confirmed. These results support previous studies that showed how coping strategies played a crucial role when people were facing an adverse situation [34,35,39,40]. They also suggest that perception and resource factors do not operate in “a vacuum” while influencing Polish healthcare personnel’s psychological well-being; they are strongly interconnected with coping processes. It highlights the interplay of cognition (risk perception) and motivation (meaning-based resources) in managing stressful events and contributing to successful adaptation to stressors [51]. Problem-focused and meaning-focused coping is thus a dynamic process that varies according to one’s cognitive appraisal and personal resources, and, consequently, regulates psychological well-being.

The present study builds on existing findings by revealing the cognitive and motivational mechanisms that underlie the effect of risk perception on psychological well-being in Polish healthcare personnel as they combat COVID-19. The two mediating strategies are predominately based on cognitive (problem-focused coping) and motivational (meaning-focused coping) processes. In contrast, emotional coping proved to be a less significant way of coping with stress during the pandemic. The utility of cognitive coping strategies in dealing with COVID-19 pandemic was confirmed by Gerhold’s research [39], which showed that Germans tend to use problem-focused strategies in coping with COVID-19 (e.g., following expert advice and guidelines and thinking carefully about what to do). The current study extends Gerhold’s [39] observations, by demonstrating that both problem-focused and meaning-focused coping operate simultaneously as mediators between risk perception and psychological well-being as well as between meaning-based resources and psychological well-being. There is, therefore, a noticeable interplay of cognitive and motivational processes underlying coping mechanisms in dealing with the dangers posed by COVID-19.

These findings suggest that although risk perception of the COVID-19 infection is related to lower psychological well-being, it paradoxically increases coping strategies, particularly problem-focused and meaning-focused coping (i.e., actions that help control the epidemics). In this sense, as noted by Li, Yang, Dou, Wang and colleagues [49], perceived severity can be regarded as a double-edged sword, being both risk and asset, in the encounter of pandemic.

These results can be interpreted within PMT, which posits that people confronted with threats resort to coping strategies that allow them to manage the threat. The decision to employ coping strategies is a consequence of severity, because people must believe that there is some potential harm (e.g., a high probability of infection with COVID-19 for healthcare personnel), and that they are vulnerable to this harm. The perception of threat from COVID-19 enhances the motivation to initiate the coping process [7]. The ability to adopt the recommended coping response may in turn enhance psychological well-being. This is in line with the meta-analysis of PMT findings by Milne et al. [52], which indicated that threat perception made it more likely that people would adopt some coping response because it provided motivational energy.

Our study shows that higher meaning-based resources predicted a more frequent use of problem-focused and meaning-focused coping, which was then related to a higher level of psychological well-being. Thus, personal resources that reflect the individual’s convictions about having a significant, meaningful, and valuable life tend to play an important role in shaping coping behavior and, consequently, in predicting the well-being of healthcare personnel. These results can be interpreted within resource-based stress theories, which suggest that individual resources influence people’s abilities to manage stress successfully and, consequently, to improve their well-being [12]. Meaning-based resources may be conducive to a constructive use of coping strategies, since a relatively stable pattern of commitment influences the way events are perceived and managed [25]. The predictive role of meaning-based resources in shaping the coping behavior of healthcare personnel is consistent with previous reports regarding the role of meaning in life in strengthening people’s coping behavior and influencing their psychological well-being [19,20].

The present study also provides new empirical evidence by demonstrating that risk perception and meaning-based resources can operate together in predicting psychological well-being in healthcare personnel combating COVID-19. Problem-focused and meaning-focused coping mediated both the relationship between risk perception of COVID-19 infection and psychological well-being as well as the relationship between meaning-based resources and psychological well-being. However, the effect test showed that the mediating path for meaning-based resources was significantly stronger than for risk perception. This implies a more important role for coping strategies based on cognitive and motivational processes for healthcare personnel well-being in the case of personal meaning-based resources than the perceived risk of COVID-19 [20,38]. However, the mere availability of meaning-based resources does not contribute automatically to well-being, these resources must rely on coping strategies based on cognitive and motivational processes.

The present study has shortcomings. It should be emphasized that the cross-sectional design limits our ability to make a causal interpretation of the findings. The relationship between risk perception and well-being as well as the relationship between personal resources and well-being can be bilateral; risk perception and personal resources can affect healthcare personnel’s well-being, but well-being may also contribute to more positive appraisals and higher meaning-based resources. Second, our explanation of the results of mediation analysis must be treated with caution. The model we tested, (which was confirmed), was based on theories and previous empirical research, but it should also be tested using a longitudinal design. Third, although respondents completed the measures anonymously, response bias could not be controlled, because the study was based on self-report. Therefore, future researchers need to attempt replication of the results with samples where this weakness is minimized.

## 5. Conclusions

Despite these limitations, the present study demonstrates the value of examining the mediating role of coping in the relationship of risk perception and personal resources with psychological well-being amongst healthcare personnel. Risk perception of COVID-19 infection negatively correlated with psychological well-being, whereas meaning-based resources had a positive correlation with well-being. Both risk perception and meaning-based resources were indirectly related to psychological well-being through the mediating effect of two coping strategies—problem-focused and meaning-focused coping. Higher risk assessment and more meaning-based resources predict more frequent use of problem-focused and meaning-focused coping, which, in turn, is related to a higher level of psychological well-being. The results contribute to a process-oriented approach towards adjustment to adverse situations [20,37,38] amongst healthcare personnel who are often on the frontline in combating COVID-19. They can also provide ideas for the creation of new meaning-based intervention programs that target existential distress and psychological well-being; analogical programs have been found very beneficial in healthcare settings [53,54]. As healthcare workers tend to experience intense stress, fatigue, and anxiety, meaning-based programs can help them find additional sources of meaning in their lives related to e.g., family, goals, values, or personal strengths. It will very likely strengthen the workers’ resilience, reduce stress, and increase awareness of professional relationships, which, in turn, will result in higher psychological well-being.

## Figures and Tables

**Table 1:** Summary and definitions of the key constructs included in the study.

Construct	Definitions
Risk perception of COVID-19 (Predictor)	
Risk of contracting	Perceived probability of being infected by COVID-19.
Fear	Fear of COVID-19.
Perceived threat	Perceived harmfulness of the consequences of being infected by COVID-19.
Meaning-based resources (Predictor)	
Meaning in life	The extent to which one’s life is experienced as making sense, directed and motivated by valued goals.
Mattering	One’s experiences of value, worth, and transcending everyday life conditions.
Coping strategies (Mediators)	
Problem focused	Efforts to obtain information about what to do and how to alter the stressful situation.
Emotion focused	Efforts to regulate the emotional distress associated with the situation.
Meaning focused	Changing the appraised meaning of a situation to make it more consistent with individuals’ beliefs and goals.
Psychological well-being (Outcome)	Positive psychological functioning and human development.

**Table 2:** Means, standard deviations, and correlations for risk perception of COVID-19, meaning-based resources, and psychological well-being.

	Variables	M	SD	1.	2.	3.	4.	5.	6.	7.	8.	9.
1.	Risk	4.19	0.63	-								
2.	Fear	3.69	0.87	0.74 ***	-							
3.	Threat	4.44	0.52	0.60 ***	0.57 ***	-						
4.	MIL	5.24	1.18	0.08	0.06	0.11	-					
5.	EM	4.64	0.71	0.02	0.11	0.06	0.39 ***	-				
6.	Problem	3.61	0.57	0.20 **	0.14 **	0.06	0.40 ***	0.49 ***	-			
7.	Emotion	3.40	0.65	0.29 ***	0.36 ***	0.23 ***	0.24 ***	0.37 ***	0.44 ***	-		
8.	Meaning	3.66	0.63	0.21 **	0.22 ***	0.17 ***	0.41 ***	0.62 ***	0.66 ***	0.62 ***	-	
9.	PWB	3.71	0.73	−0.18 **	−0.18 **	−0.16 *	0.51 ***	0.31 ***	0.42 ***	0.20 **	0.29 ***	-

* *p* < 0.05; ** *p* < 0.01; *** *p* < 0.001. Risk—risk of contracting COVID-19; fear—fear of COVID-19; threat—perceived threat of COVID-19; MIL—meaning in life; EM—existential mattering; problem—problem-focused coping; emotion—emotion-focused coping; meaning—meaning-focused coping; PWB—psychological well-being.

**Table 3:** Results of the confirmatory factor analysis: factor loadings (standardized values).

Latent Factors with Indicators	Estimate (Standardised)	SE	*p*
Risk perception of COVID-19			
1. Risk of contracting COVID-19	0.88	0.06	<0.001
2. Fear of COVID-19	0.85	0.06	<0.001
3. Perceived threat of COVID-19	0.71	0.05	<0.001
Meaning-based resources			
1. Meaning in life	0.82	0.06	<0.001
2. Mattering	0.70	0.05	<0.001

**Table 4:** Standardized indirect and total effects, standard errors (SE), and 95% confidence intervals (CI).

Model Pathways	Effect	SE	95% CI	
			Lower	Upper
Indirect effects				
Risk perception of COVID-19 → Coping → PWB	0.11 a	0.04	0.04	0.19
Meaning-based resources → Coping → PWB	0.44 a	0.07	0.29	0.56
Total effects				
Risk perception of COVID-19 → PWB	0.31 a	0.07	−0.46	−0.17
Meaning-based resources → PWB	0.44 a	0.07	0.29	0.56

PWB—psychological well-being; a—empirical 95% confidence interval does not overlap with zero.

## Supplementary Materials

The following are available online at https://www.mdpi.com/2077-0383/9/10/3225/s1. Table S1: The order in which the variables and paths were removed from the initial model on a basis of modification indices.
