# Supplementary material for: Risk Perception of COVID-19, Meaning-Based Resources and Psychological Well-Being amongst Healthcare Personnel: The Mediating Role of Coping
Source: J Clin Med. 2020 Oct 8;9(10):3225. doi: 10.3390/jcm9103225 (PMC7599885; doi:10.3390/jcm9103225)
Supplement: Supplementary file 1 [file jcm-09-03225-s001.pdf]

Supplementary material

The order in which the following variables and paths were removed from the initial model on a basis of modification indices:

1) The path from Meaning-based resources to Psychological well-being
2) The path from Meaning-based resources to Emotion-based coping
3) The path from Emotion-based coping to Psychological well-being
4) The variable Emotion-based coping along with its path from Risk perception
